# Sarcoidosis with oral involvement

**DOI:** 10.11604/pamj.2018.30.241.12010

**Published:** 2018-07-31

**Authors:** Hakima Elmahi, Fatima Zohra Mernissi

**Affiliations:** 1 Department of Dermatology, University Hospital Hassan II, Fes, Morocco

**Keywords:** Sarcoidosis, oral, buccal mucosa

## Image in medicine

Sarcoidosis is a multisystem granulomatous disease in which pulmonary involvement is the most characteristic feature. Even though extrapulmonary manifestations occur infrequently in the area of the head and neck, an occasional patient will have oral involvement. Oral involvement generally appears in patients with chronic multisystem sarcoidosis and seldom occurs in the acute stage. The oral lesions may be solitary, multiple or part of a generalized disease. In some cases, oral involvement is the first, or only, manifestation of the disease and appears as a nontender well-circumscribed brownish red or violaceous swelling, as papules, or as submucosal nodules that can occasionally either show superficial ulceration or be symptomatic. Gingival involvement presents as red gingival enlargement. Although the etiology of sarcoidosis is unknown. The diagnosis of sarcoidosis is established when clinical features are supported by histopathological evidence of typical non-caseating epithelioid granulomas and other laboratory tests. The differential diagnosis of oral soft tissue lesions must consider other granulomatous conditions, such as infections (tuberculosis, leprosy, tertiary syphilis, systemic mycoses, and cat-scratch disease), Crohn's disease, Melkersson-Rosenthal syndrome (including Mieschers cheilitis or cheilitis granulomatosa), Wegener's granulomatosis, foreign body reactions and hairy cell leukemia. Management of oral sarcoidosis has been variable, ranging from no treatment to radiation therapy. Surgical excision and curettage were found to be the most common therapies, followed by systemic therapy such as steroids, methotrexate, hydroxychloroquine and minocycline. We report a case of sarcoidosis limited to oral involvement in a 12-year-old girl, treated with corticosteroid.

**Figure 1:**
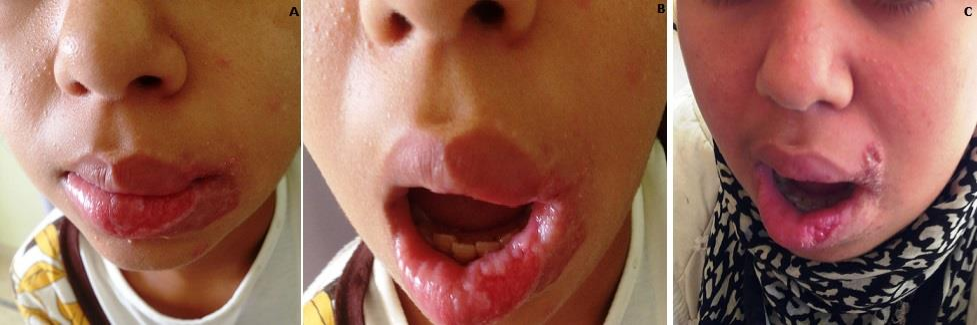
(A) clinical image of oral disease; (B) clinical image of oral disease (3/4); (C) clinical image after 6 weeks of treatment

